# Novel Human Epidermal Growth Factor Receptor 2 (HER2)-Targeted Therapy in a Non-Smoking Asian-American Woman Diagnosed With Never-Smoker Non-Small Cell Lung Cancer (NSCLC): A Case Report

**DOI:** 10.7759/cureus.106042

**Published:** 2026-03-28

**Authors:** Kyle S Yang

**Affiliations:** 1 Orthopaedic Surgery/Interventional Spine Medicine, University of California Los Angeles David Geffen School of Medicine, Los Angeles, USA

**Keywords:** asian-american women, her2 (erbb2) exon 20 insertion, never-smoker lung cancer, non–small cell lung cancer (nsclc), precision oncology, zongertinib

## Abstract

Never-smoker non-small cell lung cancer (NSCLC) represents a distinct molecular subtype enriched for actionable driver mutations, including ERBB2 (HER2) exon 20 insertions, which occur in approximately 2-4% of cases and are more common in women and individuals of Asian ancestry. We report a 75-year-old never-smoking, asymptomatic Asian-American woman with incidentally discovered stage IV (cT1cN0M1a) lung adenocarcinoma. Imaging demonstrated innumerable bilateral solid, part-solid, ground-glass, and cavitary pulmonary nodules, with the largest measuring 29 × 25 mm, without nodal or distant metastases. Lung biopsy confirmed TTF-1 and Napsin A-positive adenocarcinoma, and genomic profiling identified an ERBB2 (HER2) exon 20 insertion mutation (p.A775_G776insYVMA; VAF 3.9%). She enrolled in a clinical trial of the selective HER2 tyrosine kinase inhibitor zongertinib (60 mg twice daily) in February 2025 and tolerated therapy well, with mild diarrhea, lactose intolerance, brittle nails, and intermittent muscle cramps. Serial imaging demonstrated an early partial response by RECIST 1.1 at six weeks, followed by sustained radiographic stability with cavitary changes consistent with treatment effect. As of January 2026, she has maintained lung-confined disease control for over 11 months without extrapulmonary progression and remains fully functional without symptoms. This case highlights the clinical benefit and tolerability of selective HER2-targeted therapy in HER2-mutant NSCLC and adds to emerging evidence supporting consideration of risk-based lung cancer screening strategies in high-risk never-smoking populations.

## Introduction

Approximately 15-20% of lung cancers occur in never-smokers, representing a distinct clinical and molecular entity characterized by a predominance of adenocarcinoma and enrichment for actionable driver mutations [1]. These cancers are more common in women and individuals of Asian ancestry, with frequent alterations in EGFR, HER2, and other targetable genes [2,3]. Recent U.S. data demonstrate higher lung cancer incidence among Asian female never-smokers compared with other groups, suggesting that factors beyond smoking, such as genetic susceptibility and environmental exposures, contribute to disease risk [4].

HER2 (ERBB2) exon 20 insertion mutations occur in approximately 2-4% of NSCLC cases and are enriched in never-smokers and women [5]. Historically, treatment options for HER2-mutant NSCLC were limited, with modest responses to conventional chemotherapy. However, recent advances in HER2-targeted therapies, including antibody-drug conjugates (e.g., trastuzumab deruxtecan) and tyrosine kinase inhibitors (e.g., zongertinib), have expanded therapeutic options and improved clinical outcomes [5].

Despite these advances, current U.S. lung cancer screening guidelines remain largely based on smoking history and may not identify high-risk never-smokers, particularly among Asian women [4,6]. Risk-based screening approaches may help address this gap.

I report a case of incidentally detected stage IV (cT1cN0M1a) HER2-mutant lung adenocarcinoma in a never-smoking Asian-American woman with a sustained response to HER2-targeted therapy [7].

## Case presentation

A 75-year-old Asian-American woman with no significant past medical history underwent routine CT coronary calcium scoring for cardiovascular risk assessment. She denied cough, dyspnea, hemoptysis, chest pain, fatigue, weight loss, fever, or other constitutional symptoms. She also reported no prior pulmonary disease, environmental or occupational exposures, or recent respiratory infections. The scan incidentally revealed multiple pulmonary nodules.

**Figure 1:**
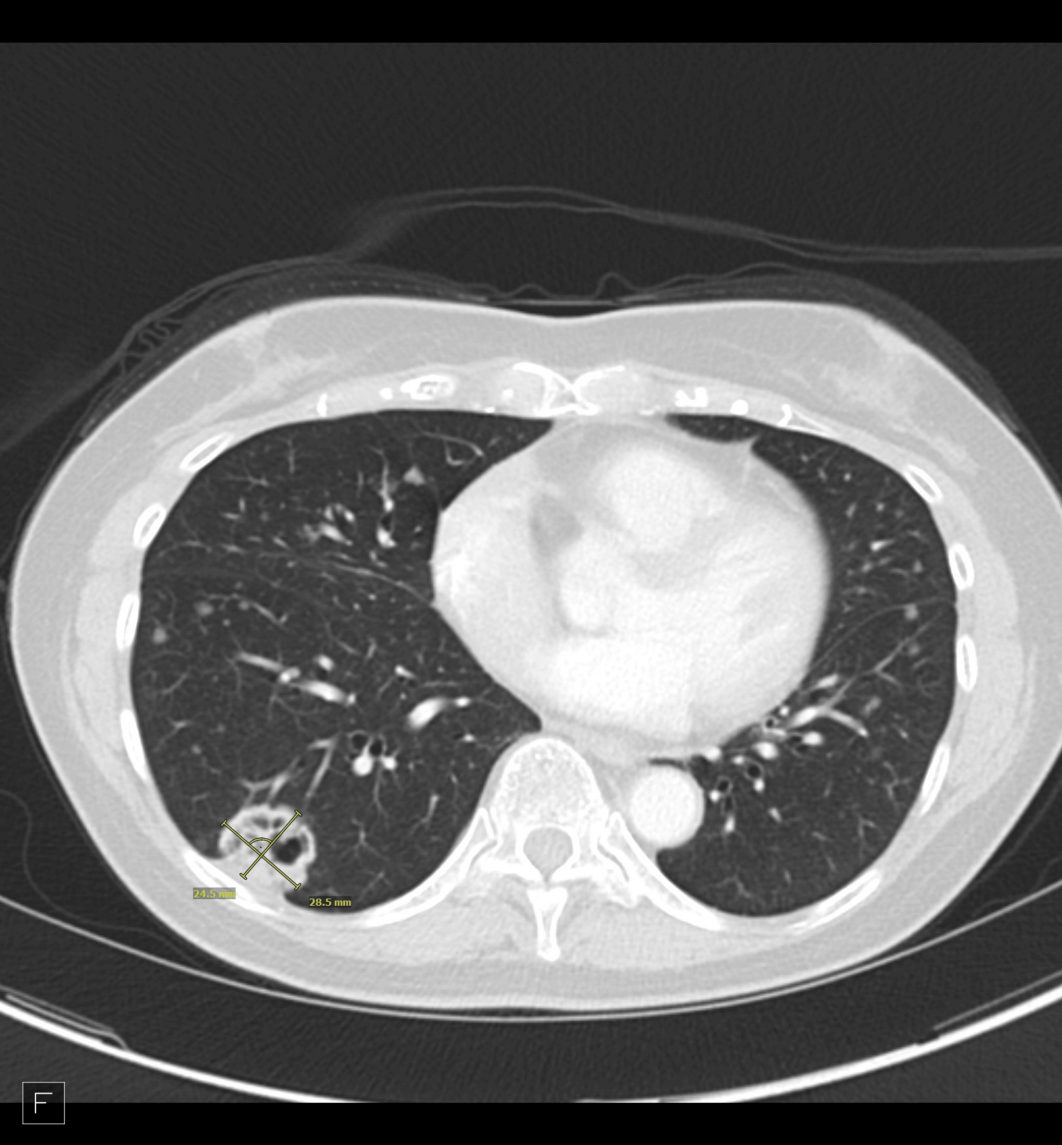
Contrast-enhanced axial chest CT demonstrating bilateral pulmonary nodules with a large cavitary lesion (measuring 29 × 25 mm) in the right lower lobe marked by a yellow X. CT: Computed tomography

Contrast-enhanced chest CT demonstrated numerous bilateral pulmonary nodules of varying morphology, including solid, part-solid, ground-glass, and cavitary (Figure 1).

No mediastinal or hilar lymphadenopathy was identified. Additionally, no evidence of extrathoracic disease was identified on CT abdomen/pelvis or brain MRI.

^18F-FDG PET/CT revealed innumerable FDG-avid pulmonary lesions without nodal or distant metastatic uptake, consistent with lung-confined metastatic dissemination (stage IV, cT1cN0M1a) according to the American Joint Committee on Cancer TNM staging system (8th edition) [7]. Despite an extensive tumor burden, the patient remained asymptomatic with an Eastern Cooperative Oncology Group (ECOG) performance status of 0.

The differential diagnosis for multifocal bilateral pulmonary nodules with mixed morphology included metastatic disease, multifocal primary lung adenocarcinoma, infectious etiologies (including atypical or fungal infections), and inflammatory conditions; however, histopathologic confirmation established the diagnosis of metastatic lung adenocarcinoma.

Video-assisted thoracoscopic biopsy confirmed pulmonary adenocarcinoma (TTF-1 and Napsin A positive). Comprehensive genomic profiling was performed using a tissue-based next-generation sequencing platform (Tempus xT), which was negative for EGFR, KRAS, BRAF, ALK, ROS1, RET, and MET alterations but identified an ERBB2 (HER2) exon 20 insertion mutation, specifically p.A775_G776insYVMA, with a variant allele frequency (VAF) of 3.9%. No concurrent actionable co-mutations were identified. Given her asymptomatic status and preference to avoid cytotoxic chemotherapy, she enrolled in a clinical trial of zongertinib, a selective HER2 tyrosine kinase inhibitor, initiated as first-line systemic therapy at a dose of 60 mg twice daily on February 13, 2025. Treatment was well tolerated, with mild diarrhea, lactose intolerance, brittle nails, and intermittent muscle cramps. Laboratory monitoring remained stable.

Serial CT imaging every six weeks demonstrated an early treatment response, as summarized in Table 1, outlining the diagnostic timeline, treatment initiation, and longitudinal imaging findings. At six weeks, dominant lesions decreased in size (right lower lobe nodule 1.7→1.3 cm and right lower lobe superior segment mass 3.3→2.5 cm), consistent with a partial response according to RECIST version 1.1 criteria [8]. Subsequent imaging from May through August 2025 demonstrated stable disease. Imaging in September 2025 demonstrated increasing cavitation of treated lesions, consistent with the treatment effect. Transient mild enlargement in late 2025 did not meet criteria for disease progression and subsequently stabilized. Follow-up imaging in January 2026 demonstrated persistent bilateral pulmonary nodules with cavitary changes and no evidence of extrapulmonary progression. Overall, the patient has maintained lung-confined disease control for more than 11 months while remaining asymptomatic and fully functional (Table 1).

**Table 1:** Timeline of diagnostic evaluation, treatment initiation, serial imaging, and RECIST 1.1 response. Chronological timeline of diagnostic evaluation, initiation of HER2-targeted therapy, and serial contrast-enhanced CT chest with concurrent CT abdomen/pelvis imaging in a 75-year-old never-smoking Asian-American woman with stage IV (cT1cN0M1a) HER2-mutated NSCLC. Early interval imaging demonstrated measurable reduction in dominant lesions, followed by durable radiographic stability with cavitary evolution consistent with treatment response. No abdominal, pelvic, or intracranial metastases were observed.

Date	Clinical Event	Imaging / Diagnostic Findings	Treatment Phase	RECIST 1.1 Assessment*
12/9/24	CT Heart Calcium Scoring	Incidental bilateral pulmonary nodules identified	Pre-treatment	N/A
12/17/24	Initial Contrast CT Chest + CT Abdomen/Pelvis (Baseline Imaging)	Largest RLL lesion 29 × 25 mm; innumerable bilateral solid, groundglass, part-solid, and cavitary nodules; no mediastinal/hilar lymphadenopathy; no abdominal/pelvic metastases	Pre-treatment	Baseline
1/6/25	Video-Assisted Thoracoscopic Biopsy	Lung adenocarcinoma confirmed	Pre-treatment	N/A
1/7/25	PET/CT	FDG-avid pulmonary nodules; no nodal/distant disease	Pre-treatment	Baseline staging
1/14/25	Brain MRI	No intracranial metastatic disease	Pre-treatment	Baseline staging
1/22/25	Tempus Molecular Testing	ERBB2 (HER2) exon 20 insertion mutation	Pre-treatment	N/A
2/13/25	Initiation of Oral Zongertinib 60mg Twice Daily	HER2-targeted therapy started	Treatment Initiated	N/A
3/27/25	Contrast CT Chest + CT Abdomen/Pelvis	Dominant lesions decreased (RLL 1.7→1.3 cm; RLL superior 3.3→2.5 cm); no new lesions	On-therapy	Partial Response (PR)
5/13/25	Contrast CT Chest + CT Abdomen/Pelvis	Stable nodules; no new lesions	On-therapy	Stable Disease (SD)
6/18/25	Contrast CT Chest + CT Abdomen/Pelvis	Stable nodules; no new lesions	On-therapy	SD
8/3/25	Contrast CT Chest + CT Abdomen/Pelvis	Stable examination; no new lesions	On-therapy	SD
9/11/25	Contrast CT Chest + CT Abdomen/Pelvis	Stable nodules with increasing cavitation; no new lesions	On-therapy	SD
10/26/25	Contrast CT Chest + CT Abdomen/Pelvis	Transient mild enlargement; no new lesions	On-therapy	SD
12/6/25	Contrast CT Chest + CT Abdomen/Pelvis	Mixed but overall controlled response; no new lesions	On-therapy	SD
1/15/26	Contrast CT Chest + CT Abdomen/Pelvis	Stable nodules with persistent cavitary changes; no abdominal/pelvic metastases	On-therapy	SD

## Discussion

This case illustrates the distinctive radiographic and molecular features of HER2-mutant NSCLC in a never-smoker. Multifocal bilateral pulmonary nodules with mixed solid, ground-glass, and cavitary components, in the absence of nodal or distant metastases, represent an uncommon but recognized presentation of lung-confined stage IV (M1a) disease. The patient’s preserved functional status despite extensive radiographic tumor burden further highlights the potential for clinical-radiologic dissociation in oncogene-driven lung cancers.

HER2 exon 20 insertion mutations result in constitutive activation of downstream signaling pathways, including PI3K/AKT and MAPK, promoting tumor proliferation and survival [5]. These mutations are enriched among women and never-smokers, reinforcing the importance of comprehensive genomic testing in this population [3,5]. In this case, genomic profiling was performed using the Tempus xT next-generation sequencing platform, identifying a HER2 exon 20 insertion (p.A775_G776insYVMA; VAF 3.9%). This variant is among the most commonly reported HER2 exon 20 insertions in NSCLC and has been associated with sensitivity to HER2-targeted therapies. No concurrent actionable co-mutations were identified. Inclusion of these molecular details enhances interpretability and facilitates comparison with emerging clinical datasets. Unlike EGFR or ALK alterations, HER2 mutations historically lacked highly effective targeted therapies, and outcomes with conventional chemotherapy were modest.

Recent advances in selective HER2-directed therapies, including antibody-drug conjugates and tyrosine kinase inhibitors, have significantly improved response rates and durability compared with historical controls [5]. In this patient, treatment consisted of the selective HER2 tyrosine kinase inhibitor zongertinib administered at a dose of 60 mg twice daily as part of a clinical trial, initiated as first-line systemic therapy in the absence of prior treatment. Therapy was well tolerated, with a favorable adverse event profile limited to mild diarrhea, lactose intolerance, brittle nails, and intermittent muscle cramps, without treatment-limiting toxicity. Targeted therapy resulted in a RECIST-defined partial response followed by sustained radiographic stability exceeding 11 months without extrapulmonary spread [8]. Cavitary evolution of treated lesions likely reflects tumor necrosis and therapeutic response rather than progression. Although positron emission tomography-computed tomography (PET/CT) confirmed lung-confined metastatic disease, quantitative metabolic parameters such as SUVmax, metabolic tumor volume (MTV), and total lesion glycolysis (TLG) were not reported in the clinical imaging dataset, limiting objective assessment of baseline metabolic tumor burden and comparison with other studies.

Notably, the patient maintained an ECOG performance status of 0 despite diffuse bilateral pulmonary involvement. This apparent clinical-radiological dissociation may be explained by several factors characteristic of oncogene-driven lung adenocarcinomas, including a predominant lepidic or minimally invasive growth pattern, relative preservation of alveolar architecture, and limited inflammatory response within the tumor microenvironment. These features may allow for substantial radiographic tumor burden without significant impairment in gas exchange or symptom burden. Additionally, the absence of nodal or extrapulmonary disease may have contributed to the patient’s preserved functional status.

While the clinical course observed in this patient is notable, it should be interpreted in the context of a single-case report, which inherently limits external validity. Clinical behavior and treatment response in HER2-mutant NSCLC can be heterogeneous across larger cohorts, and, therefore, broader conclusions regarding disease biology and therapeutic efficacy should be made cautiously. Additional studies are needed to better define the range of clinical outcomes and optimize management strategies in this molecular subset.

Beyond therapeutic implications, this case highlights critical screening considerations. Epidemiologic data demonstrate increased lung cancer incidence among Asian female never-smokers in the United States [4]. Emerging evidence suggests that low-dose CT screening in Asian female never-smokers may detect lung cancer at rates comparable to those observed in traditionally eligible smoking populations [6]. However, current U.S. screening guidelines remain based primarily on smoking history and may fail to identify high-risk never-smoking individuals. Broader risk-based screening strategies incorporating ethnicity, sex, and genetic predisposition may improve early detection and clinical outcomes, although such approaches require validation in larger prospective studies.

This case underscores three key principles: (1) NSCLC in never-smokers represents a biologically distinct disease entity; (2) comprehensive molecular profiling is essential for identifying actionable driver mutations such as HER2; and (3) selective HER2-targeted therapy can achieve durable disease control with favorable tolerability in appropriately selected patients, while recognizing the need for further validation in larger populations.

## Conclusions

We report incidentally discovered stage IV (cT1cN0M1a) HER2-mutant NSCLC in a 75-year-old, never-smoking Asian-American woman who achieved early partial response and durable lung-confined disease control with selective HER2-targeted therapy. This case reinforces the molecular distinctiveness of NSCLC in never-smokers and highlights the clinical benefit of precision oncology approaches. It also adds to emerging evidence suggesting a potential role for risk-based CT screening in high-risk never-smoking populations and underscores the need for further study in this area.
